# Dimensions of global population projections: what do we know about future population trends and structures?

**DOI:** 10.1098/rstb.2010.0133

**Published:** 2010-09-27

**Authors:** Wolfgang Lutz, Samir KC

**Affiliations:** 1 International Institute for Applied Systems Analysis (IIASA), Schlossplatz 1, A-2361 Laxenburg, Austria; 2 Austrian Academy of Sciences, Dr Ignaz Seipel-Platz 2, 1010 Vienna, Austria; 3 Vienna-University of Economics and Business 2–6 Augasse, 1090 Vienna, Austria

**Keywords:** world population, population increase, population decline, dimensions of population projections, age structure, level of educational attainment

## Abstract

The total size of the world population is likely to increase from its current 7 billion to 8–10 billion by 2050. This uncertainty is because of unknown future fertility and mortality trends in different parts of the world. But the young age structure of the population and the fact that in much of Africa and Western Asia, fertility is still very high makes an increase by at least one more billion almost certain. Virtually, all the increase will happen in the developing world. For the second half of the century, population stabilization and the onset of a decline are likely. In addition to the future size of the population, its distribution by age, sex, level of educational attainment and place of residence are of specific importance for studying future food security. The paper provides a detailed discussion of different relevant dimensions in population projections and an evaluation of the methods and assumptions used in current global population projections and in particular those produced by the United Nations and by IIASA.

## 1. Introduction

While future trends in the number and composition of humans on this planet has been a topic of scientific enquiry and discussion for centuries and at least since Thomas Malthus entered the field of structured quantitative analysis, the first modern global population projection, which explicitly considered the age and sex structure of the population (the so-called cohort-component method), was carried out by Frank Notestein of the Princeton Office of Population Research in 1945 (Notestein 1945). At the national level, several population projections precede this first comprehensive global projection. Hajnal (1955) provides a good overview of these early population projections.

Frank Notestein subsequently became the first director of the then newly established United Nations Population Division. This unit began producing regular global population projections in the early 1950s. Between 1951 and 2008, the UN published 21 sets of estimates (past and current conditions) and projections (future) for all countries and territories of the world. Before 1978 these projections were revised approximately every 5 years; since then new revisions (called assessments and published in their *World population prospects* series) have been made every 2 years. The most recent UN assessments have a time horizon to 2050. Bongaarts (2009) gives a concise summary of the main results and implications of the UN projections. At irregular intervals the UN also publishes long-term population projections with time horizons from 2150 to 2300.

The World Bank started to produce independent population projections in 1978. These were always meant primarily for internal use in the Bank's development planning and were published as part of the series of *World development reports*. After 1984, the World Bank projections were revised approximately every two years and in most cases only one variant was published but with a long time horizon to 2150. Around 1995, the World Bank stopped publishing separate projections but presumably continued to use them for internal purposes for a number of years. The Washington-based Population Reference Bureau (PRB) publishes independent world population projections (population size only and a single scenario) every year as part of its annual *World population data sheet*. Since 2000 it has published projected population sizes for all countries and territories of the world for 2025 and 2050. In some cases the projections are identical to those of the UN and the US Census Bureau (USCB), but in some cases different country-specific information is used.

The USCB produces single scenario projections for all countries in the world as of 1985 with a varying time horizon. The World Population Programme of the International Institute for Applied Systems Analysis (IIASA) based outside Vienna (Austria) began producing global population projections at the level of 13 world regions in 1994. One of the purposes was to produce population projections as part of the Special Report on Emissions Scenarios (SRES) (Nakicenovic *et al*. 2000) that underlie the global emission scenarios used by the Intergovernmental Panel for Climate Change (IPCC). This was followed by three rounds of probabilistic projections at the level of 13 regions (which were all published in the pages of *Nature*: Lutz *et al*. 1997, 2001, 2008*b*). The IIASA projections come from a distinctly scientific background, which is illustrated by the fact that in its publication (e.g. Lutz *et al*. 2004*a*) much more space is used for justifying and discussing methods and assumptions than for presenting results. Recently, IIASA developed (in collaboration with Eurostat and the UK Office of National Statistics) a new approach for evaluating substantive arguments about alternative assumptions on possible future trends based on large numbers of questionnaires ascertaining the evaluation of alternative arguments by experts. This is currently being translated (together with Oxford University) into a new set of science-based population projections for all countries in the world by age, sex and level of education for publication in 2012.

O'Neill *et al*. (2001) published a comprehensive 85-page review entitled *A guide to global population projections*. In this article, they describe in considerable detail the projections of all five agencies discussed above. The paper includes a critical review of the methods used and of the ways that assumptions are defined by those five agencies. In addition, the results are compared in quite some detail. For this reason, we do not want to repeat the exercise of systematically comparing the approaches and results of the different projections, but rather focus on a few key challenges that point to the future.

In the next section we will discuss the important questions of what we call ‘dimensions’ of population projections. The basic idea is that any population is an assembly of individuals and each individual is different from any other individual. But which of the many relevant properties of individuals should we explicitly consider in population projections? The lowest dimensionality is chosen for the case that all individuals are taken as equal (homogeneous) and only a total growth rate is assumed for projecting population size. The cohort-component method breaks down by age and sex; this is a first step in the direction of increasing dimensionality by explicitly considering these individual properties. But there are many further properties that deserve consideration in population projections and which will be discussed in the following section.

We will also address the tedious but very important issue of how to deal with uncertainty in population projections. While a single best-guess population projection is likely to satisfy the expectations of a majority of users, disregarding the issues of uncertainty can be rather dangerous and therefore deserves serious attention. We will then turn to the one issue that has recently received the greatest public attention, namely, the questions if and when we will see an end of population growth followed by a possible population decline. In the final section of the paper we propose a specific existing population projection (the UN scenario of the IIASA Education Projections) for use in the Foresight Project. We present the results for the specified regions and countries in tabular and graphical form.

## 2. Dimensions considered in population projections

For many users the most important piece of information expected from population projections is the future total size of the population; for many policy and research questions other dimensions of the composition of the population are also of interest. The dimensions that will be explicitly discussed here are age, sex, rural/urban place of residence, educational attainment, labour force participation, parity status, household status and health status. All these dimensions have in common that they are properties of each individual member of the population which are, at least in theory, observable and measurable. They can hence be called observable sources of population heterogeneity. These dimensions are not only interesting in their own right for helping to answer specific questions relating to their future distributions in the population but to the extent that they are associated with different fertility, mortality and migration intensities, their changing distributions in the total population also impact on the projected future population size. In the following, we will discuss each of these dimensions individually, assess to what degree they are likely to impact on the course of overall population, discuss how they may be important in their own right in the context of studying food security, and review existing global level projections, which explicitly address this dimension.

It is important to note that in addition to these observable sources of population heterogeneity, there is still unobserved heterogeneity in every population which is hard to capture empirically. Theoretical considerations suggest that such unobserved heterogeneity can significantly impact future population dynamics (Vaupel & Yashin 1985), but there is little one can do about it except to be aware of the problem and be cautious about the validity of the conclusions drawn. Given this problem associated with hidden heterogeneity, it is even more important to explicitly measure and incorporate the observable sources of population heterogeneity wherever feasible and thus try to minimize the possible biases caused by overall heterogeneity.

### (a) Population size

Most users of population projections seem to be primarily interested in the total size of the population. From a substantive point of view, however, it is difficult to think of specific policy or research questions, where only changes in absolute size matter and where it is supposedly irrelevant whether one refers to newborn babies, young adults or frail elderly people. Presumably, the great interest in data on the sheer number of people has two main reasons. First, total population size can be seen as a first-order approximation of the scale of the population-related issues in particular, if it is assumed that the age patterns of the two populations to be compared do not differ greatly. Second, total population size is an important denominator of many frequently used indicators ranging from gross domestic product (GDP) *per capita* to food consumption per person to greenhouse gas emissions per person. In all those cases a separately derived total quantity is divided by total population size in order to produce an indicator that can be compared across populations.

Traditionally, population projections have been produced by simply taking total population size and making assumptions on the future growth rates of the population. This has been the standard method until the age-specific, so-called cohort-component method became the widely used standard after World War II. But for some specific applications, where the age structure is difficult to assess or where the population is simply too small to have meaningful age groups (as is sometimes the case with small area population projections), simple growth rate-based projections are still being used. But for national population projections, age-group-wise projections have clearly become the state-of-the-art because they allow differentiation between the behavioural components (fertility, mortality and migration) and embedded changes that are merely owing to age-structural effects. This is particularly important for countries that have gone through significant fluctuations in fertility and mortality trends and hence have irregular age structures, such as most European countries after the two world wars. But even for high fertility countries that traditionally had very stable and regular age pyramids, the assumed future fertility decline will be partly compensated in terms of population growth through the age-structural momentum, i.e. the fact that in the future larger numbers of potential mothers will enter the reproductive age and the number of births will continue to increase even if period fertility is at replacement level. This is another good reason for explicitly considering the age structure wherever possible.

### (b) Age and sex

Particularly in the context of population ageing, the explicit interest in the population's changing age structure has recently increased significantly. While there has always been an interest by planners in the likely future number of school-age children or young adults trying to enter the labour market, the currently dominating questions relate to the sustainability of pension systems under conditions of rapid increase in the number of elderly. Closely related questions deal with the likely impact of ageing on healthcare cost or care for the elderly in general. There is significant concern that an increase in the mean age of the labour force may be associated with decreasing productivity in the future (Skirbekk 2008). These are all questions for which a projection of the population by age is essential. Much of this discussion, however, is based on fixed age categories and does not consider the fact that healthy life-expectancy tends to increase in parallel with total life-expectancy. Hence, a 65-year-old person today is typically quite different from one of the same age who lived several decades ago and who had a much shorter remaining life-expectancy. This idea has recently been translated into a redefinition of age, where age is not just measured as the time since birth but can alternatively be viewed as the expected time to death, in which case the population dynamics looks quite different (Lutz *et al*. 2008*b*; Sanderson & Scherbov 2008).

The distinction between men and women as a key dimension of population dynamics has been a feature of demography almost from the beginning. It has to do with the fact that fertility rates are almost exclusively measured with women. One of the reasons for this is that not all fathers are known and that the reproductive age range for women is shorter than for men and shows a clearer and partly biologically determined age pattern. Also, mortality rates tend to differ significantly between men and women. Except for some cases of extreme female discrimination, male mortality rates are typically higher at every single age. In addition to these demographic reasons there is, of course, significant substantive interest in the age-specific proportions of men and women in the population which matters for issues ranging from the marriage market to labour market to consumer preferences.

The formal model for doing population projections by age and sex was introduced by Cannan (1895) but it took until after World War II to have this cohort-component method spread around the world as the dominant way for doing population projections. Today, it is used by all national and international statistical agencies.

### (c) Rural–urban place of residence

Place of residence has always been an important dimension since the very origin of demography when statistical information on households was collected for taxation purposes and conscriptions for the army. Associating people with a specific locality, however, requires that people have a more or less permanent residence and are not permanently on the move. This is one problem facing statisticians when trying to distinguish between urban and rural populations in countries (such as China), where millions of people (the so-called floating population) are constantly on the move between their home villages and the urban centres where they work but have no legal right of residence. Another problem with comparing reported proportions urban across countries and over time is that there is a multitude of changing national sets of definitions based partly on the size of the agglomeration, its statutory status, its population density or the proportion of the labour force that is working in agriculture. Since urbanization will be discussed elsewhere, it will not be covered here.

From a population projections point of view, the question whether a person lives in a rural or an urban community is not only seen in its own right but is also viewed as a relevant source of population heterogeneity. Almost universally women in urban areas have lower fertility than women in rural areas. Also in terms of international migration, new immigrants today generally move to urban areas except for some special cases where immigrant labour is concentrated in agriculture. With respect to mortality, the pattern is more complex. While the inhabitants of cities traditionally had higher mortality owing to a higher risk of infectious disease, this pattern virtually disappeared over the course of the twentieth century because healthcare and education were better in urban areas. But most recently there are indications of a reversal, with urban slums in some African cities having worse mortality conditions than even remote villages in the same country.

From a methodological point of view, the best model to project a population by age and sex for urban and rural areas separately is the so-called multi-state model, which was developed at IIASA in the 1980s (Rogers & Land 1982; Keyfitz 1985). It has become the state-of-the-art for multi-dimensional population projections in which separate fertility, mortality and migration rates are assumed for the different sub-populations as well as age- and sex-specific transition rates between sub-populations. Because of data limitations, such true multi-state projections by urban–rural place of residence exist only for selected groups of countries.

The United Nations Population Division publishes the only urbanization projections on a global level, with updates every two years. The most recent revision (United Nations 2008) estimates the population living in urban areas in 229 countries and territories of the world for the period 1950–2005 and projects the same for 2010–2050 in five year intervals. The basic method of estimation and projection is still the same as when it was developed in the 1970s with only a few minor modifications. The main source of information used in the projection is the urban–rural growth difference at the two latest available time points (mostly inter-census). The difference is then assumed to follow a logistic path and is estimated based on the past experience of many countries and the expectation that the urbanization process will slow down as the level of urbanization grows. In other words, this is not a multi-state model, but only the application of assumed overall proportions urban to national level projections. This model by definition assumes continuous increases in the proportion urban and is unable to model possible ups and downs. This model is isomorphic to the literacy projection model that has been used by UNESCO for decades, but which was recently replaced by a cohort-component model.

### (d) Educational attainment

Like the other dimensions discussed above, education is also an important source of population heterogeneity and bears a significant weight of its own. Almost universally more educated people have lower mortality and there is sufficient evidence that this is a real effect and not just owing to selectivity. Also for all populations that are still in the process of demographic transition, more educated women have lower fertility. These educational differentials can be very significant. The Demographic and Health Survey for Ethiopia, for instance, shows that women without any formal education have on average six children, whereas those with secondary education have only two (see http://www.measuredhs.com). Significant differentials can be found in most populations of all cultural traditions. Only in a few modern societies does the strongly negative association give way to a U-shaped pattern in which the most educated women have a somewhat higher fertility than those with intermediate education. But globally, the education differentials are so pervasive that education may well be called the single most important observable source of population heterogeneity after age and sex (Lutz *et al*. 1999). There are good reasons to assume that during the course of a demographic transition the fact that higher education leads to lower fertility is a true causal mechanism, where education facilitates better access to and information about family planning and most importantly leads to a change in attitude in which ‘quantity’ of children is replaced by ‘quality’, i.e. couples want to have fewer children with better life chances.

In terms of measuring education, it is important to distinguish between stock and flow variables. Most education programmes and studies are concerned with the process of schooling itself, including many important topics that range from the construction of schools and the training of teachers to the organization of schools, the quality of teaching and the content of curricula. The extent to which these schooling efforts cover the entire population are usually measured through enrolment rates as published for most countries by UNESCO and other agencies. But for many of the social and economic benefits of education it does not directly matter how many people are in school at any given point in time, but rather how many have completed their education and use their acquired skills in the labour force. Hence, the main interest is not on the flow (school enrolment) but rather on the changing stock (human capital). The stock can be measured by categories of highest educational attainment (here the UNESCO-ISCED categories have become the standard) as well as by mean years of schooling. Since the original empirical data mostly come in terms of attainment and the calculation of mean years of schooling requires additional country-specific assumptions, the former is often preferred. Also, considering the full attainment distribution provides information about inequalities that is hidden in the case of only using an average such as the mean years of schooling.

Because of the strong association between female education and fertility, future changes in the composition of the female population by educational attainment make a big difference. Since many developing countries have seen major improvements in school enrolment rates of both girls and boys in those countries, the future women of reproductive age are bound to be more educated than today's. This great momentum and rather easy predictability of the educational attainment distribution is a consequence of the fact that education is typically acquired early in life and then remains virtually constant along cohort lines. If we know how many 10-year-old girls with some primary education there are today, we know (when considering differential mortality and migration) how many 50-year-olds with at least primary education there will be in 40 years' time. Given the fact that in most countries the younger cohorts are better educated than the older ones, further declines in fertility as well as mortality are virtually pre-programmed. In a few African countries where school enrolment rates have actually declined over the past two decades, this has led to a stalled fertility decline and partly worsening mortality conditions.

Aside from its effects on population dynamics, there is also significant interest in knowing the educational structure of the population for a broad range of social and economic development concerns. Based on a newly reconstructed set of educational attainment distributions by age and sex (Lutz *et al*. 2007*a*) for most countries back to 1970, it has recently been shown that indeed the improvement of educational attainment in the working age population has been the most consistent and significant driver of economic growth around the world (Lutz *et al*. 2008*a*). But improving education by age and sex has also been shown to matter for countries transitioning to democracy and more liberal rights (Abbasi-Shavazi *et al*. 2008; Lutz 2009*b*). It has already been mentioned that education is perhaps the single most important health determinant. For the question of food security, it has long been shown that the basic education of the agricultural labour force is a key factor in agricultural production (Hayami & Ruttan 1971). As the set of population–education–development–agriculture (PEDA) models commissioned by the UN Economic Commission for Africa for a number of African countries shows, when including education in an agricultural production function, it turns out to be one of the key determinants in reducing malnutrition and food insecurity (Lutz *et al*. 2004*b*).

IIASA has recently produced population projections by age, sex and four levels of educational attainment for 120 countries following different scenario assumptions with respect to alternative future education trends and different education-specific fertility trends (KC *et al*. 2010). These projections are based on a true multi-state model, i.e. individuals are assumed to be subjected to different mortality and fertility levels depending on their educational attainment level. Hence, even identical trends in education-specific fertility can lead to different overall fertility levels in the case of differing education trends. We will return to this issue when discussing the recommended combination of these education projections with the UN population projections by age and sex.

### (e) Other relevant dimensions

There are several other dimensions of population that are considered relevant either in their own right or as further important sources of heterogeneity. The parity distribution of the female population, i.e. the distribution of women by the number of children they have already given birth to, is an important determinant of fertility in the near-term future. Highly important for economic and labour force consideration is the distribution of the population by age, sex and labour force participation rates. While there are several national projections of future labour force participation, consistent global projections do not yet exist. Another very relevant dimension in the context of global population ageing and the associated needs for care for the elderly is the projection of the population by health status. While significant progress has been made in producing internationally comparable indicators of disability and its impact on activities of daily life, up to now this has not yet led to the production of global projections which cross-classify health status with age and sex. Alternatively, one could try to classify people according to certain disease risks or infection statuses (such as HIV-positive) if the data are available, and a certain disease is of specific interest. Finally, an important field of analysis and projections deals with the living arrangements/household composition of people. Here, some progress in the direction of global projections has recently been made in the context of trying to project future greenhouse gas emissions and the importance of demographic/household factors in climate change mitigation (van Imhoff & Keilman 1991; Jennings *et al*. 2004; Jiang & O'Neill 2009).

## 3. Treatment of uncertainty

In the field of social and economic trends—as in all fields that deal with human behaviour—the future can never be projected with certainty. In the context of the cohort-component model of population projection that differentiates by age and sex, all three components of population change (i.e. future fertility, mortality and migration trends) are uncertain. For each of these three components of change, the levels as well as the specific age patterns (by sex) are uncertain, but the level is considered to be of primary importance.

*How to deal with uncertainty in population forecasting* was the topic of a recent special issue of the *International statistical review* (Lutz & Goldstein 2004). It summarizes the state-of-the-art in a field of research that has recently gained more interest both among demographers as well as users. Based on this analysis, we will distinguish between four different ways that are commonly used to deal with the difficult issue of uncertainty: (i) ignore uncertainty and publish only one projection; (ii) define alternative probability-free scenarios; (iii) publish high, medium and low variants that are supposed to cover a ‘plausible range’; and (iv) produce fully probabilistic projections that give quantitative information about the range of uncertainty.

For many users it is sufficient to have only one ‘best-guess’ forecast. This is the projection which is seen as the most likely population trajectory from today's perspective, based on the best knowledge available today. Such a projection can give some orientation about the direction into which things are moving. It is sufficient for many applications with a relatively short time horizon and where the costs associated with a deviation from the projected mean are negligible. But for many planning purposes these costs can be rather significant. For example, if there is a plan to build a new primary school and the projection should tell the local authorities how many 5–10-year-olds there are likely to be in the next 10–20 years in a certain school district, such projections are associated with significant uncertainties and any deviations from the best-guess projection can turn out to be rather costly. This is even more so for projections of the number of people with pension entitlements in a given country. In terms of global level projections, those presented by the USCB, the World Bank and the Population Reference Bureau provide only single projections for all countries in the world.

The definition of ‘alternative scenarios’ is a frequently chosen approach that goes beyond a single projection and presents the user with several possible future trajectories. The terminology is somewhat inconsistent and variants (see next paragraph) are often called scenarios. Generally, scenarios are defined as consistent sets of assumptions. The notion originally came from the world of theatre, where scenes show consistent pictures of our possible setting. In demography, this is typically done by combining different assumptions about future trends in fertility, mortality and migration in a way that should ‘not be impossible’. Hence, scenarios are generally considered free of probabilities with the only criterion being internal consistency. The user is given a broad range of what could be theoretically possible without being told what is likely to happen and what probability range is covered by the interval between the highest and lowest scenario considered. At the global level, the most frequently used population scenarios are those produced in the context of the SRES scenarios of the IPCC (Nakicenovic *et al*. 2000), which in turn are based on combinations of projections produced by IIASA and the United Nations Population Division. Eurostat also presents its population projections in the form of different scenarios and an increasing number of European national statistical agencies follow this practice.

The presentation of ‘high, medium and low variants’ goes a step further in providing the user with some range of uncertainty that can be interpreted intuitively. For many decades, this approach has been the common practice of the UN population projections as well as a large majority of national statistical offices. The variants are mostly generated by assuming three alternative fertility trajectories, which are then combined with identical mortality and migration trajectories. They are typically presented to the user as providing a ‘plausible range’ of future population trends, hence providing a first approach to uncertainty although the user is not specifically told what is meant by ‘plausible’. Does it mean 100 per cent of all possible cases, 80 per cent or only 50 per cent? A further shortcoming of this approach is that it only covers the uncertainty associated with future fertility trends and does not include uncertainties associated with future mortality or migration. Since fertility is the most important determinant of long-term population growth, this can be viewed as an acceptable simplification when interested primarily in total population size. When the interest is in population ageing, however, or age-specific indicators such as the future proportion of the population above age 80, this can result in a major underestimation of uncertainty. A final problem with this approach arises when aggregating national projections to regional or global ones. The global high variant of the UN projections is calculated as the sum of all national level high variants, hence assuming that there is a perfect correlation among all national trends. Each country simultaneously experiences the highest plausible fertility trajectory. Such perfect correlation is highly unlikely, however, and a higher than expected trajectory in one part of the world may be partly offset by diverging trends in other parts of the world. Hence, what is considered as a plausible range at the national level is not necessarily plausible at the global level.

Only ‘fully probabilistic’ projections can avoid such problems. These are based on pre-defined uncertainty distributions over time for each of the three components which are stochastically combined in large numbers (typically several thousands) of cohort-component projections with the individual random draws being subject to assumed autocorrelations and, in the case of multi-regional projections, correlations among the regions. In most projections, the components are assumed to be uncorrelated between each other. As the field of probabilistic population projections has grown, there have been essentially three traditions in defining the uncertainty distributions: one based on time series analysis (which is only applicable to countries that have long empirical series of data and where no structural discontinuities are expected); one based on the analysis of the errors of old projections (*ex post* error analysis combined with the assumption that future errors will essentially be the same as past projection errors); and one based on the evaluation of expert arguments resulting in sets of subjective probability distributions. While more experts as well as national and international agencies have entered this field of probabilistic projections, some convergence among these different approaches seems to be ongoing. It is also interesting that the initiative for doing such projections does not always come from the producers but rather from the users. For example, in the case of the UK Office of National Statistics, such projections were recently produced upon request by the financial planning authorities. At the global level, IIASA (Lutz *et al*. 1997, 2001, 2008*b*) has produced the only probabilistic population projections at the level of 13 world regions.

From a theoretical perspective, fully probabilistic projections are superior in avoiding the problems of other approaches as discussed above. They provide the users with the most comprehensive and detailed information in terms of the demographic ‘risks’ that can be combined with a cost function. But this approach is criticized by some as conveying a false sense of precision. It is indeed true that this approach requires more detailed explicit assumptions in terms of the full distributions and correlations than the other common approaches. Yet, the proponents of probabilistic projections argue that they only try to make otherwise implicit assumptions explicit and extensive sensitivity analyses show that the overall results tend to be very robust to changes in the specific shapes of the assumed uncertainty distributions. But at this stage it is clear that a universally accepted approach to quantitatively describing the uncertainty of population projections has not yet been developed and more research in this field is needed.

A final source of uncertainty that often tends to be disregarded in population projections is the uncertainty about current conditions, i.e. the population size and structure as well as the levels of fertility and mortality in a given country in the starting year of the projections. While this tends to be well documented in industrialized countries, there are major gaps of information, in particular, in Africa and parts of Asia. For many countries the information is only based on sample surveys and for some countries even such information is not available. While the UN Population Division makes heroic efforts to come up with population estimates for all countries in the world, the fact that many numbers referring to the recent past have to be adjusted in every new assessment round as new information becomes available shows how difficult this task is. But demographers have become so used to having a precise point estimate for any demographic indicator, even in countries with very fragmentary empirical information, that they often forget about the uncertainty of those indicators referring to the starting conditions when making projections. A recent study on past projections in five southeast Asian countries (Khan & Lutz 2008) showed that the errors introduced by incorrect information about starting conditions was in some cases even higher than the error owing to incorrect assumptions about the future. Lutz *et al*. (2007*b*) expanded the concept of probabilistic population projections to explicitly include the uncertainty about starting conditions in addition to the uncertain future paths for the three components. This is even relevant at the level of world population projections because of the great uncertainty of current fertility levels in the world's most populous country, China, as will be discussed in more detail below.

## 4. The prospect of world population stabilization or decline after peak

There has been much discussion recently about the long-term outlook for world population and in particular whether the global population is likely to stabilize or even decline in size after a peak during the second half of the twenty-first century. This discussion was, in part, triggered by an article published in *Nature* in 2001 entitled *The end of world population growth* (Lutz *et al*. 2001), which indicated that there was an 80–90% chance that the world population would reach a peak before 2100 and start to decline thereafter. Whether or not such a peak will actually occur and at what level and how steep the following decline will be is primarily a function of the assumptions made about long-term fertility levels in different parts of the world.

When it comes to global population projections beyond 2050, there are only the occasionally published UN long-term projections as well as the IIASA world population projections for comparison. In 2004, the UN Population Division published a study entitled *World population to 2300* (United Nations 2004) in which different long-term scenarios were presented for all countries in the world by extending their usual projections to 2050 by another 250 years. While life-expectancy was assumed to continue to increase over the entire period although at a decelerating rate, the fertility scenarios defined were all very close to replacement level. Of the three long-term fertility assumptions, the low one assumed universal convergence to 1.85, the high one to 2.35 and the medium one to whatever the replacement level might be (somewhat below 2.1 under good mortality conditions). It is interesting to note that the fertility level of 1.85, which in the projections to 2050 is assumed to be the convergence level of the medium variant (with the low variant being 1.35) for the long-range projections, is the lowest fertility scenario presented. But even these very small differences in the long-term fertility levels produce significantly different long-term global population sizes: by 2100, the resulting populations are 5.5 (low), 9.1 (medium) and 14.0 (high) billion, and by 2200, the differences further increase to 3.2, 8.5 and 21.2 billion, respectively. Hence, the medium scenario results in some sort of population stabilization in the very long run, but only because global long-term fertility is assumed to remain constant exactly at the replacement level of fertility—a level that is defined as the one producing long-term stationarity. Minor deviations to the lower side will produce significant population decline and to the higher side will result in substantial long-term increases.

**Figure 1.**
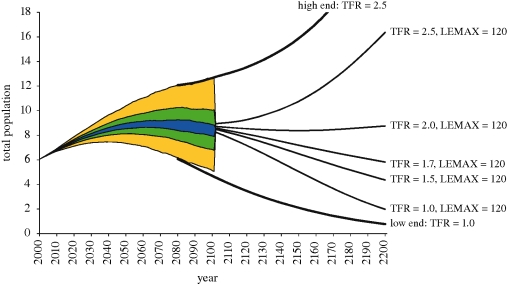
Total world population in billions: probabilistic projections until 2100. Yellow, 95% interval; green, 60%; blue, 20% and extensions to 2200. The scenarios shown combine different levels of total fertility rate as indicated with the assumption that life-expectancy continues to increase up to a maximum level of 120 years. Source: Lutz & Scherbov (2008).

Lutz & Scherbov (2008) recently published another set of long-term global projections, which extends the IIASA probabilistic population projections—which go to 2100—further into the future by defining scenarios covering a wider range of possible future fertility levels. Selected findings are shown in figure 1 and were discussed in a recently published editorial in the *Journal of the Royal Statistical Society* entitled *Towards a world of 2–6 billion well-educated and therefore healthy and wealthy people* (Lutz 2009*a*). The extension scenarios start in 2080 (the year until which the assumptions for the probabilistic projections were defined); some scenarios continue from the level of the median of the projected distribution; others from the upper and lower bounds of the projected 90 per cent uncertainty range (figure 1).

The figure clearly illustrates what has been discussed above with regard to the UN long-range projections. In the long run global fertility levels below 2.0 will result in population decline and above 2.0 in long-term population increase. But the figure also illustrates that there is a real chance that global population could fall below its current size by the middle of the next century, even if global fertility levels were somewhat higher than what is being experienced in Europe today.

## 5. Proposal for use of population projections in food security assessments

The criteria for recommending a specific population projection to be used for the assessment and planning of global food security include both the widespread use and acceptance by major international agencies as well as substantive dimensions, such as the detail of relevant information, its scientific basis and the plausibility of the assumptions made.

In terms of the use of international population projections, there is no doubt that the UN population projections dominate the field. This is mostly owing to their long and well-established tradition and the easy availability of country-specific data for both estimates and projections (from 1950 to 2050). Also, for all international agencies that are part of the UN family of agencies (including the Food and Agriculture Organization), there is an institutional agreement to only and consistently use the UN projections in order to avoid embarrassment arising from using different numbers in different parts of the UN. Hence, despite the above described shortcomings of the UN projections, particularly in the way they deal with uncertainty, there is no doubt that virtually all groups and agencies dealing with food security and agriculture expect to see the UN medium variant as their population projection of choice. For this reason and with the only exception of China—as discussed below—this driver review also recommends using the UN medium variant in terms of population size and the age and sex structures that come with it.

But this review can do better than just considering the population by age and sex. As discussed above, education is a key dimension in the study of development and food security. IIASA has recently produced population projections for most countries in the world by age, sex and four levels of educational attainment up to 2050. While the baseline scenario in these projections deviates from the UN medium variant in assuming different future fertility levels throughout Europe (following the Eurostat projections that have been worked out in collaboration with national statistical offices) as well as in some east Asian countries, IIASA has also calculated a so-called ‘UN Scenario’ for comparative purposes. Since in the projections by level of education assumptions are defined in terms of future education-specific fertility and mortality trends, which then have to be weighted by the size of the respective education categories if overall fertility and mortality rates are to be calculated, the changing educational composition over time induces differences to the UN projections even if education-specific fertility and mortality assumptions are defined to be as close as possible to the UN assumptions. A full congruence of the two sets of projections can only be achieved through an iterative procedure in which for every country and every point in time, the education-specific rates are modified in a way that their weighted average becomes identical to the overall rates assumed by the UN (2009). This procedure has been performed for all countries for the ‘UN-Scenario’ and results in a projected age, sex and education structure that is identical to the age and sex structure of the UN medium variant, but also gives the education distribution as well. The user can then choose whether to use this scenario, which is perfectly in line with the widely used UN projections, or alternatively take the projections that result from the independently defined education-specific fertility trends. Since for the coming 40 years (time horizon 2050), the differences between these two scenarios are minimal, in the following we will illustrate only the ‘UN Scenario’.

Only for China do we recommend a deviation from the UN medium variant because China has such significant weight when studying the world population and there are convincing arguments that fertility in China is currently significantly lower than given by the UN and is likely to stay so for the coming decades. The 2008 UN assessment gives a total fertility of 1.77 for China for the periods 2000–2005 and 2005–2010. This is subsequently assumed to increase to 1.79 in 2010–2015, 1.84 in 2015–2020 and stay constant at 1.85 thereafter. But the level of fertility in China around the 2000 census and thereafter has become a topic of intense scientific analysis and discussion. While the National Statistical Agency published the figure of 1.22 for the census year, most scholars agree that this number reflects an undercount with the only question being how much of an undercount. In an effort to produce probabilistic population projections for China that also assume uncertain starting conditions, Lutz *et al*. (2007*b*) reviewed more than 20 different estimates based partly on different methods and different data sources. They concluded that the best guess for 2000 was a level around 1.5 with significant uncertainty bounds. Most recently, a new study by Morgan *et al*. (2009) convincingly demonstrates that total fertility is currently slightly below 1.5 and is expected to remain there at least for the coming two decades. Based on this strong scientific reasoning, we suggest that the best-guess projections used for China should be based on a constant total fertility assumption of 1.5. This has been implemented in the output tables and graphs given below.

## 6. Description of results

The following section presents and discusses selected results of the chosen UN Scenario of the IIASA global projections by level of education by using the regional definitions given as a standard for all the driver reviews. These projections are available at the level of individual countries on the website of IIASA's World Population Programme (www.iiasa.ac.at/Research/POP). In addition to six continents they list the data for four key countries and five regions of special interest. This scenario was calculated on the basis of the 2006 UN assessment before the 2008 assessment came out, and the UN made minor adjustments between the 2006 and the 2008 revisions. Therefore, small discrepancies might appear between the data listed here and those currently available from the website of the UN Population Division.

Table 1 gives the results in terms of total population size. It shows the total world population increasing from an estimated 6.885 billion in 2010 to 7.6 billion in 2020, 8.2 billion in 2030 and around 9 billion by 2050. These numbers clearly indicate the projected decelerating speed of world population growth. While the decadal increase in world population is estimated by the UN to be 760 million between 2000 and 2010, it is projected to decline to 616 million for the decade 2020–2030 and 322 million for 2040–2050. The distribution of this growth over continents shows that the population of Africa is still expected to roughly double, whereas that for Europe is already on a declining trajectory. It is worthwhile noting that the devastating AIDS pandemic, which lowered life-expectancy in the worst-hit countries and also had a minor depressing effect on population growth, does not really influence this big picture of population growth. Of the countries that are individually listed, China—currently the most populous country in the world with 1.3 billion inhabitants—will continue to grow until around 2030 owing to population momentum (i.e. more young women entering the reproductive ages) even though fertility is assumed to be well below the replacement level. By 2050, China's population size is expected to be lower than it is today and 420 million lower than India's, which is likely to surpass China as the most populous country shortly before 2020.

**Table 1.** Projections of total population size for continents as well as selected countries and regions (UN Scenario of IIASA education projections).

area	2000	2010	2020	2030	2040	2050
world	6124	6885	7617	8233	8699	9021
Africa	821	1032	1271	1518	1765	1998
Asia	3705	4145	4546	4846	5024	5095
Europe	729	730	722	707	687	664
Latin America and Caribbean	523	594	660	713	750	769
North America	316	349	379	405	427	445
Oceania	31	35	39	43	46	49
Brazil	174	199	220	236	248	254
China	1270	1330	1371	1374	1324	1238
India	1046	1220	1379	1506	1597	1658
UK	59	62	64	66	68	69
European Union	482	495	498	496	489	479
Former Soviet Union	289	284	279	271	261	249
NW Europe	246	253	258	262	262	261
Nile catchment	225	285	354	424	492	555
sub-Saharan Africa	680	867	1081	1308	1540	1761

While the picture of future population growth is quite differentiated, with some countries and regions expected to grow substantially, whereas others are expected to shrink in terms of future population ageing, all countries and regions are moving in the same direction. Currently about 8 per cent of the total world population is above the age of 65. This proportion is likely to double over the coming 20 years and by 2040 reach the level of 16 per cent, which is the level currently experienced in Europe. Asia is the most rapidly ageing continent where the current proportion above age 65 is likely to increase by a factor of three from currently 7 to 21 per cent in 2050. China will rapidly catch up with Europe and reach some 27 per cent above the age of 65 by the middle of the century, although currently its proportion elderly is only half of the European one. Even in Africa where the population structures are still very young (only 3% of the population are above age 65), the projected increase in life-expectancy together with declines in fertility will result in significant ageing in the longer run (table 2).

**Table 2.** Projections of the proportions of the population above age 65 for continents as well as selected countries and regions (UN Scenario of IIASA education projections).

area	2000 (%)	2010 (%)	2020 (%)	2030 (%)	2040 (%)	2050 (%)
world	7	8	10	13	16	19
Africa	3	3	4	5	5	7
Asia	6	7	9	13	17	21
Europe	15	16	19	23	25	28
Latin America and Caribbean	6	7	9	12	15	19
North America	12	13	16	20	21	21
Oceania	10	11	14	16	18	19
Brazil	5	7	9	13	16	19
China	7	8	12	17	24	27
India	5	5	7	9	11	14
UK	16	17	19	22	24	24
European Union	16	17	20	24	27	29
Former Soviet Union	11	11	13	16	18	21
Nile catchment	3	4	4	5	6	8
NW Europe	16	18	21	24	26	26
sub-Saharan Africa	3	3	3	4	5	6

When interpreting these numbers on projected proportions elderly, we also need to consider that disability-free life-expectancy so far tends to increase at roughly the same speed as total life-expectancy and that the 65-year-olds of the future can be expected to be in better health conditions than the 65-year-olds today. It has recently been demonstrated (Lutz *et al*. 2008*b*) that when age is not defined as the time since birth but alternatively as the expected time to death, then the coming speed of population ageing (i.e. people moving closer to their death) will be much more moderate. The future elderly are also likely to be better educated and more likely to continue to be gainfully employed depending on the incentive structures that will be in place. The greatest challenge associated with population ageing is probably in the poorest countries where often no old-age support systems exist aside from one's own family. This also needs to be considered in the context of studying future rural populations and the agricultural work force.

As discussed above, almost universally more educated people are in better health and are more productive. Recent studies have shown that there are some thresholds both with respect to health and to economic growth in the sense that universal primary education (one of the key Millennium Development Goals) is not sufficient but that it requires high proportions of the population with at least completed junior secondary education (to age 15) to help bring countries out of the vicious circle of poverty, high population growth and food insecurity (Lutz *et al*. 2008*a*). For this reason table 3 focuses on the proportion of the population with junior secondary or higher education.

**Table 3.** Projections of the proportions of the population (above age 15) that have junior secondary or higher education for continents as well as selected countries and regions (UN Scenario of IIASA education projections).

proportion with at least secondary education							
sex	area	2000 (%)	2010 (%)	2020 (%)	2030 (%)	2040 (%)	2050 (%)
female	world	53	59	65	71	77	82
	Africa	26	35	43	52	60	67
	Asia	45	54	62	69	76	82
	Europe	85	89	92	94	95	96
	Latin America and Caribbean	53	62	70	78	84	89
	North America	95	94	94	94	94	95
	Oceania	96	99	100	100	100	100
	Brazil	52	62	71	80	86	91
	China	56	66	75	82	89	93
	India	28	38	48	58	67	75
	UK	73	81	87	90	92	94
	European Union	80	85	90	93	94	95
	Former Soviet Union	96	98	99	99	99	99
	Nile catchment	25	34	42	52	60	67
	NW Europe	83	87	91	93	94	95
	sub-Saharan Africa	21	29	38	47	55	63
male	world	62	67	72	76	79	83
	Africa	38	45	52	58	63	68
	Asia	59	66	72	76	81	84
	Europe	86	89	92	93	94	96
	Latin America and Caribbean	52	60	67	73	79	83
	North America	94	94	94	94	95	95
	Oceania	96	98	100	100	100	100
	Brazil	48	56	64	72	78	83
	China	71	78	84	87	91	94
	India	47	55	63	70	75	80
	UK	73	81	86	89	91	92
	European Union	82	86	89	92	93	94
	Former Soviet Union	96	98	98	99	99	99
	Nile catchment	37	43	50	56	61	66
	NW Europe	84	87	90	93	94	95
	sub-Saharan Africa	33	39	47	54	59	65

The trends shown in table 3 are based on the Global Education Trend Scenario (KC *et al*. 2010), which assumes that in terms of the proportions of cohorts ending up in the different educational attainment categories, the countries later in the process follow the trend of the more advanced countries. Since the 1970s, this trend has been dominated by the speed of educational expansion of many Asian countries. Similar expansions are also assumed for the currently worse-educated populations in Africa which hence might be viewed as a rather optimistic scenario. Table 3 also shows that while today almost universally adult men are better educated than adult women, this is likely to change in the future because of the fact that female school enrolment rates in most countries are approaching those of men and in many countries even surpassing them.

**Figure 2.**
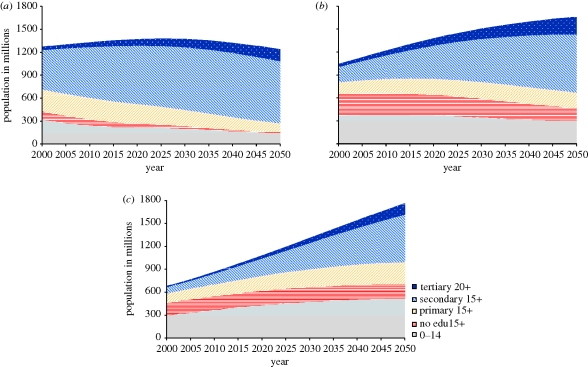
(*a*) China, (*b*) India and (*c*) sub-Saharan Africa: projected trends in the total population by level of highest educational attainment (children below age 15 in grey at the bottom).

Figure 2 shows the projected trends in the absolute numbers of the population by four educational attainment categories for India and China as well as the region of sub-Saharan Africa. The population below the age of 15 is indicated as a separate group at the bottom of the graph. While for China the picture shows a peaking in the size of the population followed by a decline over the coming decades, the number of people with secondary or tertiary education will continue to increase. The trend in sub-Saharan Africa shows a very different picture characterized by continued rapid population growth. India is in an intermediate position with decelerating population growth associated with a rapid expansion of the more educated segments of population. But even in Africa this projection of populations by level of education gives rise to more optimism for the future than the usual focus on population size alone, because it shows that the most rapidly growing segment of the population is that with secondary or tertiary education under this admittedly rather optimistic scenario.

The comparison between India and China in figure 2 is particularly interesting since they are the two population billionaires frequently mentioned together as the two great economic powers of the future. But the figures illustrate quite clearly that in terms of the human capital of their populations, the two countries are very different. Over the past decades, China has heavily invested in universal primary and near universal secondary education. Although still less than half of the total population today has secondary or higher education, this is certain to change as the better educated younger cohorts move up to higher age groups. In contrast, India suffers from the fact that currently still about half of all adult women have never been to school. This is also the main reason why fertility in India is still rather high and as a consequence the population will experience significant growth over the coming decades. Recently, school enrolment rates in India have increased at all levels but it will take many decades until India will be able to match the level of schooling of the average Chinese. This will have implications for food security, health, economic growth and adaptive capacity to climate change.

In conclusion, this review has attempted to highlight some recent developments in the methodology and the content of global population projections and in particular place emphasis on the different dimensions of population change that should be explicitly addressed in population projections. The educational attainment distribution has been singled out as a key dimension, which perhaps should be routinely added to age and sex in our studies of the trends and consequences of human population size and structures.
